# Etiology and Management of Dyslipidemia in Patients With Cancer

**DOI:** 10.3389/fcvm.2022.892335

**Published:** 2022-04-25

**Authors:** Mikhail de Jesus, Turab Mohammed, Meghana Singh, John G. Tiu, Agnes S. Kim

**Affiliations:** ^1^Department of Medicine, University of Connecticut School of Medicine, Farmington, CT, United States; ^2^Department of Medicine, Calhoun Cardiology Center, University of Connecticut School of Medicine, Farmington, CT, United States

**Keywords:** dyslipidemia, cholesterol, cancer, cardiovascular risk reduction, cancer survivor

## Abstract

Patients with cancer are now living longer than ever before due to the growth and expansion of highly effective antineoplastic therapies. Many of these patients face additional health challenges, of which cardiovascular disease (CVD) is the leading contributor to morbidity and mortality. CVD and cancer share common biological mechanisms and risk factors, including lipid abnormalities. A better understanding of the relationship between lipid metabolism and cancer can reveal strategies for cancer prevention and CVD risk reduction. Several anticancer treatments adversely affect lipid levels, increasing triglycerides and/or LDL-cholesterol. The traditional CVD risk assessment tools do not include cancer-specific parameters and may underestimate the true long-term CVD risk in this patient population. Statins are the mainstay of therapy in both primary and secondary CVD prevention. The role of non-statin therapies, including ezetimibe, PCSK9 inhibitors, bempedoic acid and icosapent ethyl in the management of lipid disorders in patients with cancer remains largely unknown. A contemporary cancer patient needs a personalized comprehensive cardiovascular assessment, management of lipid abnormalities, and prevention of late CVD to achieve optimal overall outcomes.

## Introduction

The development of highly effective anticancer therapies over the past few decades has favorably changed the landscape of patients with cancer, who can now achieve high cure rates in early stages of disease and long-term remission in others (1). This oncologic progress, however, has generated a unique patient population who are at a high risk of experiencing a myriad of chronic comorbidities, among which CVD is one of the most important (2). Cancer and CVD share several common risk factors, including advanced age, chronic inflammation, obesity, hyperlipidemia, poor diet, smoking history, and physical inactivity (3, 4). A multi-disciplinary team comprising of primary care, oncology, pharmacy, and cardio-oncology is best poised to serve this special cohort of patients who often pose challenging diagnostic and management dilemmas (5).

Dyslipidemia has been associated with poor outcomes in patients with cancer by promoting tumor invasion and metastasis (6), producing resistance to cancer drugs (7), and enhancing the cardiac and vascular toxicity of anticancer therapies (8). In this review, we discuss the emerging literature on the relationship between lipid abnormalities and carcinogenesis, review anticancer treatment-associated hyperlipidemia, discuss CVD risk assessment and risk reduction in patients with cancer, and highlight the current evidence to support the use of antilipidemic agents in this special patient population.

## Hyperlipidemia, Metabolic Syndrome, and Cancer

It is well known that dyslipidemia is a strong predictor of CVD (9, 10). Emerging data suggest that hyperlipidemia may also play a role in carcinogenesis (11). Tumor cells have been shown to require large amounts of sterol metabolites to sustain rapid growth and proliferation (12, 13). A key regulatory transcriptional factor in lipid synthesis and uptake, sterol regulatory element-binding protein (SREBP), has been identified to be dysregulated in various cancer types to accelerate endogenous cholesterol and fatty acid production (14, 15). Another mechanism reported in prostate cancer is reduced cholesterol efflux through ABCA1 (ATP-binding cassette class A) transporters (16). Additional pathways connecting cholesterol and cancer are phosphatidylinositol 3-kinase (PI3-K)/Akt pathways that are part of hedgehog signaling, which when dysregulated can lead to abnormal cell proliferation and tumor growth (11).

Higher levels of saturated and monounsaturated phospholipids in cell membranes have been shown to protect cancer cells from oxidative damage (17). Lipids also serve an important role in cell signaling and migration, as well as post-translational modification of proteins (18, 19). Angiogenesis, a hallmark of cancer, occurs through the secretion of prostaglandin E2, a sterol compound in breast cancer cells (18, 20). All of these functions highlight the importance of lipids in oncogenesis and tumor spread.

Hyperlipidemia is a common comorbidity among cancer patients and survivors. Ray and Husain demonstrated that patients with breast cancer had significant elevations in plasma total cholesterol (TC), low density lipoprotein (LDL)-cholesterol, and triglyceride (TG) levels (21). Shah et al. reported similar findings in patients with breast cancer when compared to patients with benign breast disease (22). In a large cross-sectional study, there was a significant difference in the lipid profiles among different types of cancers (23). Patients with ovarian cancer were observed to have the highest serum TG levels, while those with colorectal cancer had the lowest TG (23). Breast cancer patients had the highest TC and LDL levels, while gastric cancer patients had the lowest values (23). Interestingly, serum LDL levels greater than 110 mg/dL correlated with lymphatic metastasis (23).

Not only hyperlipidemia but also metabolic syndrome (MetS) has been associated with the development of cancer (24). Within the United States, nearly 33% of all adults and about 50% of adults older than 60 have MetS (25). In a systematic review and meta-analysis of 43 studies including 38,940 cancer cases, metabolic syndrome was found to be associated with an increased risk of several cancers including colorectal, liver, pancreas, endometrial, and postmenopausal breast cancers (26). Survivors of childhood cancer (e.g., acute lymphoblastic leukemia) were observed to have roughly two-fold higher prevalence of MetS compared with general adult population (27). Obesity, a key component of MetS, has also been identified as a risk factor for developing cancer (28, 29).

A significant association between MetS and all-cause cancer mortality has been documented in a prospective study, where MetS was associated with a 56% greater age-adjusted risk for cancer mortality (30). Women with breast cancer and MetS had a higher incidence of partial response to therapy, and high blood sugar levels were predictive of a poor response to therapy (31). The American Society of Clinical Oncology has identified obesity as one of the most important determinants of cancer mortality (28, 29).

Statins may play a role in reducing the risk of cancer development and/or progression. Lochhead et al. described the benefits of statin therapy for colorectal cancer patients (32). Patients who used statins for more than 3 years prior to their colorectal cancer diagnosis had a lower tumor stage, lower prevalence of metastasis, and higher five-year cancer-specific survival compared with statin non-users (32). There is also preclinical evidence that statins may directly block the adhesion and migration processes of cancer cells, supporting the anti-carcinogenic potential of statins (33). Anti-angiogenic effect of statins has also been reported in patients with chronic liver disease (34). Statins have been shown to induce apoptosis of hepatoma cells, inhibit intrahepatic angiogenesis, and interfere with tumor cell adhesion in hepatocellular carcinoma (34). A meta-analysis of 26 studies found that long-term statin use may reduce the risk of pancreatic cancer incidence (35). Ahern et al. reviewed the basic science and epidemiologic evidence that statins, particularly simvastatin, may reduce the risk of breast cancer recurrence. They described the broad range of existing literature that supports the anticancer effects of statins and the protective effect of statins on breast cancer prognosis (36).

## Anticancer Therapies That Have the Potential to Cause Dyslipidemia

Various drugs used in cancer therapy have been associated with lipid abnormalities, either due to chemotherapy-related gonadal failure or as a direct adverse effect of the medication (Table 1). The National Cancer Institute (NCI) classifies the severity of hypertriglyceridemia and hypercholesterolemia resulting from cancer drugs as categorized in Table 2 (37).

**Table 1:** List of anticancer therapies associated with dyslipidemia, their adverse effects on lipid profile, and the proposed mechanisms of dyslipidemia.

**Anticancer therapy**	**Effects on lipid panel**	**Proposed mechanism of dyslipidemia**
Androgen deprivation therapy	↑ TC, ↑ LDL	Gonadal failure (32, 33)
Antiestrogen therapy	↑ TC	Unknown
Anthracycline	↑ LDL, ↓ HDL	Downregulates PPAR gamma nuclear receptors and decreases apo A1 levels (38)
Tyrosine kinase inhibitors	↑ TG	Unknown
Lorlatinib (ALK TKI)	↑ TC, ↑ TG	Unknown
mTOR inhibitor	↑ TC, ↑ TG	Increases apo CIII, suppressing LPL activity and reduces clearance of VLDL (45)
VEGF Inhibitor	↑ TG	Unknown
L- asparaginase	↑ TG	Increases apo CIII and decreases apo CII, inhibits activity of LPL (51)
JAK 1/2 inhibitor	↑ TC, ↑ LDL, ↑ TG	Unknown
Bexarotene	↑ TC, ↑ TG	Unknown
Capecitabine	↑ TG	Unknown

*TC, total cholesterol; LDL, low-density lipoprotein; HDL, high-density lipoprotein; TG, triglycerides; apo CIII, apolipoprotein CIII; apo CII, apolipoprotein CII. ↑ indicates “increases the level”; ↓ indicates “decreases the level”*.

**Table 2:** National Cancer Institute (NCI) grading of hypertriglyceridemia and hypercholesterolemia secondary to anti-neoplastic agents.

**Severity of adverse event**	**Hypertriglyceridemia**	**Hypercholesterolemia**
Grade 1	150–300 mg/dL	>ULN–300 mg/dL
Grade 2	300–500 mg/dL	300–400 mg/dL
Grade 3	500–1000 mg/dL	400–500 mg/dL
Grade 4	>1000mg/dL	>500 mg/dL
Grade 5	Death	Death

## Dyslipidemia From Gonadal Failure

Various combinations of anticancer agents can lead to gonadal failure. Tian et al. examined lipid levels of over 800 patients with early-stage breast cancer in a retrospective study, during and after neoadjuvant or adjuvant chemotherapy and compared them to those of patients who underwent surgery-only therapy without any chemotherapy (38). They found that in individuals receiving chemotherapy, the serum TC, LDL and TG levels increased significantly during chemotherapy treatment, but returned to pre-chemotherapy range about 6 months after completion of therapy (38). There were no differences between the groups receiving different combination of chemotherapy regimens. In a subgroup analysis, it was noted that younger premenopausal women were more prone to dyslipidemia while receiving chemotherapy (38). A similar study also demonstrated that premenopausal women had greater alterations in their lipid panel compared to post-menopausal women (39). This difference could be attributed to changes in lipid levels from a sudden drop in estrogen secondary to chemotherapy-induced ovarian failure (39).

Similarly, in a retrospective analysis, patients with metastatic testicular cancer receiving cisplatin-based chemotherapy were noted to have an increase in TC and LDL levels, along with increased subcutaneous fat deposition and insulin resistance (40). It was also noted that the serum estradiol level was increased in these patients which could contribute to partial hypogonadism, which in turn would affect fat and glucose metabolism (40).

## Androgen Deprivation Therapy (ADT)

ADT, including gonadotropin releasing hormones (GnRH) agonists and antagonists, is the mainstay of treatment for prostate cancer (41). They inhibit the production of endogenous testosterone, causing various metabolic effects (41).

GnRH agonists (leuprolide, gosarelin) stimulate the GnRH receptor continuously, resulting in downregulation of the receptor with reduction in luteinizing hormone (LH) and subsequently testosterone levels. In contrast, GnRH antagonists (degarelix) block the same receptors and reduce the release of LH, which in turn reduces the production of testosterone (42). Anti-androgen medications like bicalutaminde and flutamide block the androgen receptors and inhibit dihydrotestosterone (DHT) from binding to it. Abiraterone acetate is an oral agent that blocks testosterone production by inhibiting the cytochrome P enzyme, CYP17 (42). ADT can cause significant changes in lipid profiles.

In a prospective study by Torimoto et al., 39 patients with prostate cancer on ADT were followed for 12 months while on therapy, with serial monitoring of their body composition and lipid levels (43). There was consistent elevation of TC and LDL levels documented throughout the year on ADT (43). Similar findings were reported by Salvador and colleagues during a 6-month follow up in patients on ADT for prostate cancer (44). They also observed no difference in the lipid profile abnormality among patients receiving GnRH agonists or bicalutamide (44). Grossman and Zajac suggested that patients receiving ADT should have a fasting lipid profile checked prior to initiation of therapy and have serial monitoring of lipid levels every 6 months (45). The American Heart Association, American Cancer Society, and American Urological Association released a scientific advisory recommending that patients have interval follow-up within 3-6 months of ADT initiation to monitor blood pressure, lipid profile, and glucose levels (46).

## Antiestrogen Therapy

Antiestrogen therapies are primarily used in patients with estrogen receptor positive breast cancer. Tamoxifen is a selective estrogen receptor modulator that binds to estrogen receptors on tumors and suppresses effects of estrogen in the tumor (47). Aromatase inhibitors (AIs), such as anastrazole, letrozole, and exemestane, are selective nonsteroidal aromatase inhibitor that prevent the conversion of androstenedione to estrone and testosterone to estradiol. They are used to treat postmenopausal women with hormone-receptor positive breast cancer. These medications can reduce the tumor mass and delay cancer progression (47).

AIs, but not tamoxifen, have been associated with an increased risk of lipid abnormalities and cardiovascular (CV) events (48). In a meta-analysis by Amir et al., patients on AIs were found to have significantly higher odds of being diagnosed with hypercholesterolemia and CVD when compared with those on tamoxifen (48). Additionally, studies on mice have demonstrated that AIs can directly affect the endothelium and predispose to the development of atherosclerosis, findings which were also illustrated as attenuation of endothelial function in human studies (49). To date, there are no official recommendations for the management of hyperlipidemia secondary to antiestrogen therapies.

## Anthracyclines

Doxorubicin has been associated with hyperlipidemia secondary to ovarian failure. However, anecdotal evidence suggests that anthracyclines could also directly affect lipid metabolism (50). Sharma et al. longitudinally followed patients with newly diagnosed breast cancer undergoing treatment with four cycles of doxorubicin and cyclophosphamide (+/- 5-fluorouracil), followed by treatments of paclitaxel and analyzed their serial serum lipid profiles. A continual increase in LDL and decrease in HDL were documented throughout the duration of therapy. *In vitro* analysis showed that doxorubicin downregulated PPAR gamma nuclear receptors and decreased apoA1 levels, which possibly reduced the production of HDL in the liver. Long-term follow up of cholesterol levels was not performed to assess for any permanent effects on lipid metabolism (50). There are no official recommendations for the management of dyslipidemia in patients receiving anthracycline treatment.

## Tyrosine Kinase Inhibitors (TKI)

Dyslipidemia has been mentioned as a possible side effect of TKI (51). Anlotinib is a TKI targeting vascular endothelial growth factor receptor (VEGFR), fibroblast growth factor receptor (FGFR), platelet derived growth factor receptor (PDGFR), stem cell factor receptor (c-Kit), and Ret (52), and is used in the treatment of advanced non-small cell lung cancer (NSCLC) (53).

Early phase clinical trials showed a higher incidence of HTG (41 vs. 23.8%) and hypercholesterolemia (41.8 vs. 14%) in the anlotinib arm than the control arm (52, 53). The time of onset of HTG in the anlotinib group was around 20 days. Most patients were treated with fibrates to lower their triglycerides, and very few needed dose reductions of anlotinib. None required drug discontinuation because of HTG (52, 53). The mechanism for this dyslipidemia is not known.

## Lorlatinib

This third-generation TKI targets anaplastic lymphoma kinase (ALK) gene with activity against NSCLC demonstrating resistant ALK mutations (54). In the phase two trial of lorlatinib in patients with NSCLC, the most common adverse effect was hypercholesterolemia (81%) and HTG (60%) with grades 3 and 4 severity of both observed in about 15% of patients (55). The median time to onset of hyperlipidemia from treatment initiation was 15 days (55). All of the 81% of patients were started on a lipid-lowering agent. In patients with grade four hypercholesterolemia, the dose of lorlatinib was held until the cholesterol level decreased to grade two severity, and then successfully reinitiated (55).

The Canadian Cardiovascular Society (CCS) recommends checking a lipid profile at baseline, 1, 2 and 3 months after starting lorlatinib and every 3 months thereafter. They also recommend starting lipid-lowering therapy when LDL is >3.5 mmol/L (~135 mg/dL) with a goal to reduce LDL level by 50% or <2.0mmol/L (77 mg/dL). They recommend withholding lorlatinib if the total cholesterol level is above 12.92 mmol/L (~500 mg/dL), until the levels decrease. The lipid-lowering therapies recommended were pravastatin or rosuvastatin as first-line therapy and ezetimibe for second-line therapy (54). Similar first-line therapy was recommended for isolated HTG (~500 mg/dL). They also recommend holding the medication if the TG level exceeds 11.4 mmol/L (~1,000 mg/dL). Fenofibrate or omega-3 fatty acids could be utilized as second-line therapy (54).

## Mechanistic Target of Rapamycin (MTOR) Inhibitors

mTOR inhibitors (e.g., sirolimus) inhibit signaling in the phosphoinositide 3 kinase (PI3K) – Akt-mTOR pathway, which plays a key role in tumor growth and lipid metabolism. While it is a useful anti-cancer therapy and anti-rejection treatment for transplant recipients, inhibition of this pathway leads to reduced clearance of LDL in the blood causing hyperlipidemia (56).

Dyslipidemia with sirolimus use usually begins 2–4 weeks after starting therapy (57, 58). In a retrospective study of renal transplant patients on immunosuppressive regimen including sirolimus, a significant increase in TG levels and a moderate increase in the total cholesterol levels was documented (57). Morrisett et al. demonstrated return of cholesterol levels to normal within 8 weeks after discontinuation of sirolimus (58). It is hypothesized that sirolimus inhibits heparin-induced lipoprotein lipase (LPL) activity resulting in increase of apo-CIII levels, which suppresses LPL activity, hence reducing the clearance of VLDL particles (57).

Given the high incidence of this adverse effect, it is recommended to check lipid panels at baseline and then serially at every cycle for patients on mTOR inhibitors. Some experts recommend checking a fasting lipid panel weekly in early phase trials (59). It is also recommended to start statins in the first month of therapy if the patient has elevated total cholesterol or triglyceride levels (57). Lipid-lowering therapy is typically started with a goal to keep fasting TG <300 mg/dL and LDL <190 mg/dL. For patients started on lipid-lowering medication, a lipid panel should be rechecked with each cycle of therapy (59).

## VEGF/VEGFR Inhibitor

VEGF/VEGFR inhibitors lead to dyslipidemia by interfering with the mTOR pathway (60). A meta-analysis revealed that patients on VEGF/VEGFR inhibitors had a higher incidence of hyperlipidemia (41%) compared to placebo (60).

Tivozanib is a VEGFR inhibitor used in patients with renal cell carcinoma (61). In the phase Ib trial of Tivozanib among patients with renal cell cancer, 30% of the recipients of Tivozanib had elevated TG levels with a grade 3/4 degree of HTG documented in up to 11% of the patients. These patients were on a relatively higher dose of tivozanib compared to other patients suggesting a possible dose-related association with HTG (61). The management strategy recommended for Tivozanib related hyperlipidemia is similar to that for mTOR inhibitors (61).

## L-Asparaginase

L-asparaginase is used in the treatment of acute lymphoblastic leukemia in children with a well-known adverse effect of lipid abnormalities (62). Parsons et al. serially examined fasting lipid and lipoprotein levels in 38 patients diagnosed with ALL before, during and after asparaginase therapy. Nineteen percent of (7/38) patients had an elevation of TG level to higher than 1000 mg/dL that reverted back to normal at the end of 2 years following therapy (62). Further lipoprotein subclass analysis revealed a significant increase in VLDL levels from 30.5 mg/dL to 396 mg/dL during asparaginase therapy (62). The proposed mechanism is inhibition of LPL, increase in apo-CIII and decrease in apo-CII levels which all lead to an increase in serum TG-rich lipoproteins in the plasma. The onset of HTG is usually 8–14 days after asparaginase therapy (63).

It is recommended that TG levels should be checked in all patients prior to asparaginase therapy. Initiation of early conservative treatment with fibrates can prevent further increase in TG levels and reduce the risk of future complications, such as pancreatitis and sagittal sinus thrombosis (64).

## JAK1/2 Inhibitor

Ruxolitinib is an oral JAK1 and JAK2 inhibitor approved for treatment of myelofibrosis (MF) and polycythemia (PV) (65). The COMFORT -I study demonstrating the efficacy of ruxolitinib in MF also showed an increase in TC and LDL levels (66). Anecdotal reports have also described HTG manifesting as steatohepatitis and pancreatitis in patients treated with ruxolitinib (65, 67). It is recommended to monitor lipid levels after starting ruxolitinib, particularly if given in combination with sirolimus for graft-vs. host disease (65).

## Bexarotene

This retinoid compound is used in the treatment of patients with refractory cutaneous T-cell lymphoma (68). HTG within 2–4 weeks of starting therapy is a known adverse effect of bexarotene seen in up to 70% of patients secondary to a rise in the production of VLDL (69). The HTG and elevated TC levels are often reversible with discontinuation of therapy. Patients are recommended to have a baseline fasting lipid panel checked prior to starting bexarotene and thereafter be checked weekly for 2–4 weeks. If stable, it can then be checked every 8 weeks. The goal is to maintain fasting triglycerides around ~400 mg/dL. If triglyceride levels rise above 400 mg/dL, it is recommended to consider starting lipid lowering therapy like statins, and if necessary, reduce the dose or interrupt bexarotene (68).

## Capecitabine

Capecitabine is a prodrug of 5-fluorouracil (5-FU) commonly used in patients with breast and colon cancer. Multiple case reports of capecitabine-induced HTG exist in the literature (70). Dumana et al. described the case of a patient with breast cancer on capecitabine who developed HTG with levels > 9,000 mg/dL requiring lipid apheresis (71). Following discontinuation of capecitabine, the lipid levels normalized with eventual discontinuation of lipid lowering therapy (71). It has been hypothesized that this HTG may be more prominent in patients with hereditary LPL deficiency (70).

## Management of Dyslipidemia in Patients With Cancer

The initial steps for the treatment of dyslipidemia, metabolic syndrome and obesity which are highly prevalent in patients with cancer are the promotion of lifestyle changes, including modification of diet and addition of an exercise routine. A diet that emphasizes consumption of fruits, legumes, nuts, whole grains, and fish is recommended. A heart healthy diet should avoid saturated and trans fats, high sodium intake, processed meats, refined carbohydrates, and sweetened beverages (72). The ACC/AHA 2019 guidelines also recommend that adults exercise at least 150 min of moderate-intensity physical activity or 75 min of vigorous-intensity aerobic physical activity per week. All adults should decrease sedentary behavior to reduce ASCVD risk (72).

Current guidelines recommend the use of statin therapy for the primary prevention of CVD in patients with LDL>190 mg/dL, diabetes mellitus, or elevated 10-year atherosclerotic cardiovascular disease (ASCVD) risk score in patients without diabetes mellitus (73). In addition, statins are recommended for all patients with established ASCVD for secondary prevention. Patients with an intermediate (7.5% to <20%) and high (>20%) 10-year ASCVD risk scores should be considered for moderate- and high- intensity statin therapy, respectively, in addition to lifestyle changes (73). The Canadian Cardiovascular Society (CCS) updated their guidelines in 2021 to propose similar recommendations to the ACC/AHA with the key difference being that they recommend risk stratification using the Framingham Risk Score (74). The Childhood Cancer Survivor Study (CCSS) developed a risk assessment tool that predicts the risk of heart failure, ischemic heart disease, and stroke by age 50 among survivors of childhood cancer (75).

The traditional CVD risk assessment tools, such as the ACC/AHA Risk Estimator/ Pooled Cohort Equation or the Framingham Risk Score, do not include cancer-specific parameters or history of cancer treatment and thus may underestimate the true long-term CVD risk in cancer survivors. A population-based cohort study showed an increase in the medium and long-term risks of CV diseases (including heart failure, coronary artery disease, arrhythmia, stroke, and venous thromboembolism) in the survivors of various adult cancers compared with the general population (76). The increased risks were most pronounced in individuals who had exposure to chemotherapy. Multiple myeloma, lung cancer, non-Hodgkin lymphoma, and breast cancer were associated with significantly higher CVD risk compared with noncancer controls. The increased risk was most pronounced in cancer survivors with two or more CV risk factors (77).

Coronary artery calcium (CAC) scoring may provide additional risk stratification in patients with cancer (78). In a population-based cohort study that evaluated 484 patients undergoing low-dose CT for lung cancer screening, higher CAC scores were associated with an increased risk of CAD (78). The CAC Consortium developed an equation to calculate the risk of death from CVD vs. from cancer (79). They found that the mortality risk from CVD exceeded that from cancer at age 50 if the CAC score is >115 and at age 70 if the CAC sore is > 570 (79). These studies suggest the utility of CAC scoring in identifying individuals with cancer who can benefit from early preventative measures.

To further refine the prediction of CVD risk in the cancer patient, additional measures such as advanced lipid markers (lipoprotein(a), apolipoprotein B) and inflammatory markers (hs-CRP) may be of benefit. Table 3 summarizes the current tools available for cardiovascular risk stratification in cancer patients. Further research is needed to elucidate which tools are the most predictive of CVD risk in this population.

**Table 3:** List of the risk stratification tools currently available to identify patients with cancer who are at increased risk of developing late atherosclerotic CVD.

**Tools to predict atherosclerotic CVD risk in patients with cancer**
American College of Cardiology/American Heart Association ASCVD Risk Estimator/ Pooled Cohort Equation
Framingham risk score
Childhood Cancer Survivor Study Cardiovascular Risk Calculator
Coronary artery calcium scoring
Lipoprotein(a), apolipoprotein B, high sensitivity C-reactive protein

## Statins

Recommendations guiding the management of hyperlipidemia in patients who are actively undergoing, or have recently completed, cancer treatment are largely lacking. The treatment of hyperlipidemia, and the primary and secondary prevention of CVD, in patients with cancer largely follow the framework proposed by the 2019 ACC/AHA guidelines (72). The management of hypercholesterolemia focuses on lifestyle modification initially, then on the addition of statin therapy, followed by consideration of other medications, such as ezetimibe and proprotein convertase subtilisin/kexin type 9 serine protease (PCSK9) inhibitors. Given the unique characteristics of patients with cancer, including their exposure to potentially cardiotoxic cancer treatment, future research is imperative to determine the ideal strategies to reduce their long-term CV risk.

Statins have been found to have pleiotropic effects, including antioxidant, anti-inflammatory, and immunomodulatory effects, as well as atherosclerotic plaque stabilization (80). They may also have anticancer effects, as discussed in the “Hyperlipidemia, metabolic Syndrome, and cancer” section above. These pleiotropic effects support the importance of statin therapy in this patient population, in which further studies investigating its potential benefits are warranted.

## Ezetimibe

Ezetimibe is the current second line therapy for hyperlipidemia. The Simvastatin and Ezetimibe in Aortic Stenosis (SEAS) study investigated the effects of combination ezetimibe/simvastatin compared with placebo on the effects of CV events (81). Initial analyses raised concerns about ezetimibe having potential carcinogenic properties. Further sub-analyses dispelled this hypothesis and found that ezetimibe does not significantly increase the risk of cancer or overall mortality (81). Meta-analyses have demonstrated that ezetimibe has beneficial effects on CVD endpoints, including myocardial infarction and stroke, without increasing all-cause or CV mortality, nor cancer development (82, 83). The addition of ezetimibe to statin therapy has been shown to cause a greater LDL reduction than doubling the statin dose (84). Given its overall benefits and safety, ezetimibe should be the ideal second antilipidemic agent of choice for individuals with cancer who have increased CV risk.

## Other Antilipidemic Agents

PCSK9 inhibitors are novel cholesterol-lowering agents that act by attaching to the LDL receptor, reducing its degradation and thus increasing LDL clearance (85). Although the data supporting the use of PCSK9 inhibitors primarily as antilipidemic agent in patients with cancer is limited, preliminary data suggests that it may potentially assist anti-cancer therapy by boosting the effect of immunotherapy by upregulating the MHC-I expression and promoting intratumoral T-cell infiltration making the tumor more responsive to immunotherapy (86). More studies are needed to analyze its lipid-lowering activity in this specific subset of patients.

There is scant data on the use of new lipid-lowering therapies like bempedoic acid in patients with cancer. It (8-hydroxy- 2,2,14,14- tetramethylpentadecanedioic acid) is a small molecule that inhibits ATP citrate lyase, a crucial step in the synthesis of cytosolic acetyl – CoA, which is the building block in cholesterol biosynthesis (87). Currently, it serves as an alternative lipid-lowering treatment in patients intolerable of frontline agents (88).

Icosapent ethyl is another newer agent that acts by reducing hepatic production of TG. There is limited data on the use of this medication in patients with cancer. The REDUCE-IT trial, which demonstrated the CV benefits of icosapent ethyl in patients with elevated TG levels, excluded patients on tamoxifen, cyclophosphamide, and patients with life expectancy of <2 years (89). Further research is needed to better define the role of non-statin therapies, including ezetimibe, PCSK9 inhibitors, bempedoic acid and icosapent ethyl, in the management of dyslipidemia in cancer patients.

## Special Considerations for the Use of Lipid-Lowering Therapy in Patients With Cancer

There are special considerations to make when initiating patients on lipid-lowering therapy that are receiving active chemotherapy (Table 4). These include potential drug-drug interactions between dyslipidemia medications and chemotherapy, patients with liver disease, and patients that suffer adverse reactions from dyslipidemia medications.

**Table 4:** Special considerations for the use of lipid-lowering therapy in patients with cancer.

	**Special considerations and recommendations for patients with cancer**
Drug-drug interactions	•Nilotinib and ribociclib are considered moderate inhibitors of CYP3A4; lorlatinib and pexidartinib are moderate inducers of CYP3A4. •Avoid statins metabolized by CYP3A4 (simvastatin, lovastatin, and atorvastatin). •Consider replacing with safer alternatives (e.g., pravastatin or rosuvastatin). •Collaborate with Pharmacy and Hematology-Oncology for a multi-disciplinary approach. •Check pharmacy references or websites for drug-drug interactions prior to prescription.
Cancer patients with liver disease	•Pravastatin, rosuvastatin, or pitavastatin are not metabolized by the liver. •Studies have found lovastatin to be safe for patients with known liver disease.
Potential side effects of lipid-lowering therapy	•Statins: hepatotoxicity, rhabdomyolysis, immune-mediated necrotizing myopathy, myalgias •Ezetimibe: hepatocellular injury, rhabdomyolysis, myopathy, myalgias, erythema multiforme, anaphylaxis, angioedema •PCSK9 inhibitors: local site reactions •Bempedoic acid: dose-related hyperuricemia, rare tendon rupture •Icosapent ethyl: increased risk of bleeding, atrial fibrillation, and atrial flutter

## Drug-Drug Interactions

Some statins are metabolized and cleared by the liver, predisposing potential drug-drug interactions. Simvastatin, lovastatin, and atorvastatin are metabolized by cytochrome p450 3A4 (CYP3A4). Thus, they can interact with other medications that are metabolized by CYP3A4, e.g., antibiotics, antivirals, antiepileptics, calcium channel blockers, and antineoplastic agents (47). Most cases of drug interactions are reported with simvastatin likely arising from competitive effect of anti-cancer drugs on CYP3A4, resulting in hepatotoxity and rhabdomyolysis from augmentation of simvastatin through decreased clearance (90). Nilotinib and ribociclib are considered moderate inhibitors of CYP3A4; lorlatinib and pexidartinib are moderate inducers of CYP3A4 (47). In patients taking TKIs such as imatinib and dasatinib, or mitotane (used in adrenal carcinoma), hepatically metabolized statins should either be tapered to the safest tolerable dose or discontinued and replaced by safer alternatives (e.g., pravastatin or rosuvastatin) (91). Awareness of potential drug-drug interactions is critical in managing patients with cancer. Collaboration with a pharmacist and/or oncologist is important. Pharmacy references or websites that check for drug-drug interactions should be utilized prior to initiating antilipidemic medications in patients who are receiving anticancer drugs, particularly the novel targeted agents.

## Cancer Patients With Liver Disease

As mentioned previously, statins are the cornerstone therapy for ASCVD risk reduction. However, myopathy and hepatotoxicity are its known adverse effects (92). This is especially concerning among patients with cancer and liver disease. Pravastatin, rosuvastatin, and pitavastatin are not metabolized by the liver and can be used for this special subset of patients (93). Statin-induced liver injury has primarily been observed with atorvastatin and simvastatin. Studies have found lovastatin to have no increased risk of hepatotoxicity in patients with known liver disease (93). Statins have pleiotropic effects, including potential inhibitory effect on the progression of liver fibrosis to cirrhosis and hepatocellular carcinoma (93). Thus, statins that are not metabolized by the liver can be safely used in patients with concomitant cancer and liver disease.

## Potential Adverse Reactions of Lipid-Lowering Therapy

Statins are generally well-tolerated medications. In addition to hepatotoxicity, rhabdomyoloysis, immune-mediated necrotizing myopathy, and myalgias are other known adverse reactions. It is important to monitor liver function tests and test for rhabdomyolysis if patients complain of myalgias. Less serious adverse reactions to statins include nasopharyngitis and diarrhea (47). Ezetimibe is also associated with hepatocellular injury, rhabdomyolysis, myopathy, and myalgias. Postmarketing studies have also found erythema multiforme, anaphylaxis, and angioedema associated with its use (47). PCSK9 inhibitors are well-tolerated with local injection site reactions, e.g., erythema, pain, or bruising, reported as the most common adverse reaction (47). Bempedoic acid is known to have a dose-related hyperuricemic effect and rarely associated with tendon rupture (47). Icosapent ethyl has been associated with an increased risk of bleeding and increased risk of developing atrial fibrillation and atrial flutter (47).

## Conclusion

The burden of CVD is exceedingly high in patients with cancer because of a high prevalence of underlying risk factors, such as hyperlipidemia, hypertension, diabetes mellitus, and metabolic syndrome. In addition, anticancer therapies may exert cardio- and vasculo- toxic effects as well as adverse effects on lipid levels. It is essential for medical providers to be aware of these side effects and promptly institute appropriate therapy as well as other CV preventive strategies. Developing CV risk assessment tools that accurately identify cancer patients who are at an increased risk of CVD is needed. Coronary artery calcium scoring and serum markers can potentially aid with risk stratification and deserve further investigation to understand their utility in patients with cancer (Table 5). Statins are the mainstay of therapy in both primary and secondary CVD prevention as well as in the management of hyperlipidemia. The role for non-statin therapies for dyslipidemia management also need further investigation as they may contribute to overall CVD risk reduction (Table 5).

**Table 5:** Future areas of investigation for mitigating cardiovascular risk in patients with cancer.

**Areas of future investigation in the management of hyperlipidemia and CV risk reduction in patients with cancer**
1. What is the best CV risk assessment tool to identify those patients with cancer who are at an elevated risk for developing late CVD?
2. What is the role of coronary artery calcium scoring in the CV risk stratification of patients with cancer?
3. What is the utility of serum markers (lipoprotein(a), apolipoprotein B, high sensitivity CRP) in these patients?
4. What is the role of non-statin therapies, including ezetimibe, PCSK9 inhibitors, bempedoic acid and icosapent ethyl, in the management of dyslipidemia in cancer patients?

**Figure 1:**
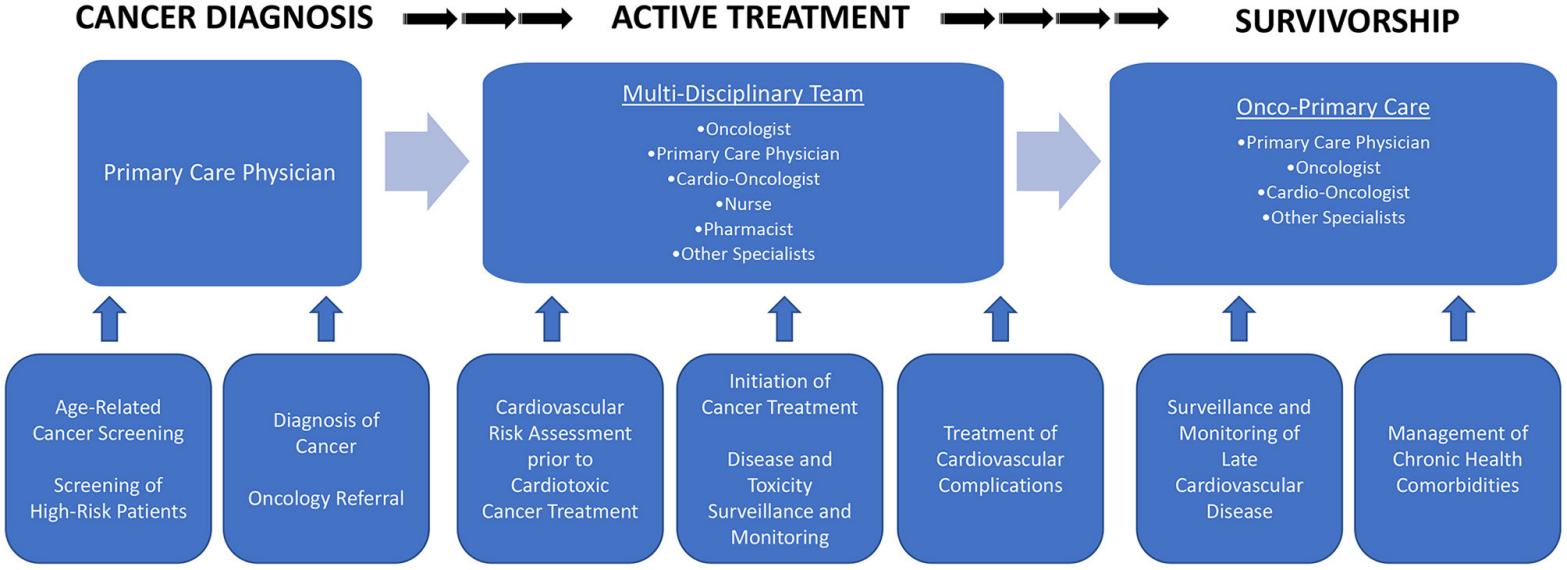
The medical journey of cancer survivors.

In summary, optimal cardiovascular care of the contemporary cancer patient requires a multidisciplinary approach to accurately define CVD risk, institute appropriate preventive measures, and address the potential adverse cardiometabolic effects of anticancer therapies. An integrative team comprised of primary care, oncology, cardio-oncology, nursing, and pharmacology devoted to the comprehensive and longitudinal care of patients from cancer diagnosis to treatment to survivorship is needed (Figure 1). This team of providers plays an integral role in cancer screening and diagnosis, monitoring for potential adverse events during cancer treatment, and management of chronic health comorbidities in survivorship (94). Understanding the myriad possible early and late side effects of cancer treatment is critical to improve overall morbidity and mortality of cancer survivors.

## Author Contributions

AK, MDJ, and TM made substantial contribution to the article design and conception of the work. MDJ, TM, MS, and JT contributed to the acquisition of data and drafting of the manuscript. AK and MDJ made major contributions to analysis, interpretation and editing of the manuscript, made critical revisions, and all authors read and approved the final manuscript.

## Funding

The funding for the article processing charge was provided by the Letts O'Brien Fund (UConn Foundation endowment fund #0035192).

## Conflict of Interest

The authors declare that the research was conducted in the absence of any commercial or financial relationships that could be construed as a potential conflict of interest.

## Publisher's Note

All claims expressed in this article are solely those of the authors and do not necessarily represent those of their affiliated organizations, or those of the publisher, the editors and the reviewers. Any product that may be evaluated in this article, or claim that may be made by its manufacturer, is not guaranteed or endorsed by the publisher.

## References

[1] Mattiuzzi C. Cancer statistics: a comparison between World Health Organization (WHO) and Global Burden of Disease (GBD). Eur J Public Health. (2020) 30:1026–7. 10.1093/eurpub/ckz216 31764976

[2] Sturgeon KM Deng L Bluethmann SM Zhou S Trifiletti D Jiang C. A population-based study of cardiovascular disease mortality risk in US cancer patients. Eur Heart J. (2019) 40:3889–97. 10.1093/eurheartj/ehz766 31761945 PMC6925383

[3] Aparecida Silveira E Vaseghi G de Carvalho Santos AS Kliemann N Masoudkabir F Noll M. Visceral Obesity and Its Shared Role in Cancer and Cardiovascular Disease: a Scoping Review of the Pathophysiology and Pharmacological Treatments. Int J Mol Sci. (2020) 21:E9042. 10.3390/ijms21239042 33261185 PMC7730690

[4] Mohammed T Singh M Tiu JG Kim AS. Etiology and management of hypertension in patients with cancer. Cardio-Oncol Lond Engl. (2021) 7:14. 10.1186/s40959-021-00101-2 33823943 PMC8022405

[5] Zamorano JL Gottfridsson C Asteggiano R Atar D Badimon L Bax JJ. The cancer patient and cardiology. Eur J Heart Fail. (2020) 22:2290–309. 10.1002/ejhf.1985 32809231 PMC8278961

[6] Luo X Cheng C Tan Z Li N Tang M Yang L. Emerging roles of lipid metabolism in cancer metastasis. Mol Cancer. (2017) 16:76. 10.1186/s12943-017-0646-3 28399876 PMC5387196

[7] Cao Y. Adipocyte and lipid metabolism in cancer drug resistance. J Clin Invest. (2019) 129:3006–17. 10.1172/JCI127201 31264969 PMC6668696

[8] Cortes JE Jean Khoury H Kantarjian H Brümmendorf TH Mauro MJ Matczak E. Long-term evaluation of cardiac and vascular toxicity in patients with Philadelphia chromosome-positive leukemias treated with bosutinib. Am J Hematol. (2016) 91:606–16. 10.1002/ajh.24360 26971533 PMC5548463

[9] Duran E Aday A Cook N Buring J Ridker P Pradhan A. Triglyceride-Rich Lipoprotein Cholesterol, Small Dense LDL Cholesterol, and Incident Cardiovascular Disease. J Am Coll Cardiol. (2020) 75:2122–35. 10.1016/j.jacc.2020.02.059 32354380 PMC8064770

[10] Collins R Reith C Emberson J Armitage J Baigent C Blackwell L. Interpretation of the evidence for the efficacy and safety of statin therapy. Lancet. (2016) 388:2532–61. 10.1016/S0140-6736(16)31357-5 27616593

[11] Brown AJ. Cholesterol, Statins and Cancer. Clin Exp Pharmacol Physiol. (2007) 34:135–41. 10.1111/j.1440-1681.2007.04565.x 17250629

[12] Schulze A Harris AL. How cancer metabolism is tuned for proliferation and vulnerable to disruption. Nature. (2012) 491:364–73. 10.1038/nature11706 23151579

[13] Röhrig F Schulze A. The multifaceted roles of fatty acid synthesis in cancer. Nat Rev Cancer. (2016) 16:732–49. 10.1038/nrc.2016.89 27658529

[14] Sun Y He W Luo M Zhou Y Chang G Ren W. SREBP1 regulates tumorigenesis and prognosis of pancreatic cancer through targeting lipid metabolism. Tumor Biol. (2015) 36:4133–41. 10.1007/s13277-015-3047-5 25589463

[15] Yin F Sharen G Yuan F Peng Y Chen R Zhou X. TIP30 regulates lipid metabolism in hepatocellular carcinoma by regulating SREBP1 through the Akt/mTOR signaling pathway. Oncogenesis. (2017) 6:e347–e347. 10.1038/oncsis.2017.49 28604762 PMC5519197

[16] Lee BH Taylor MG Robinet P Smith JD Schweitzer J Sehayek E. Dysregulation of Cholesterol Homeostasis in Human Prostate Cancer through Loss of ABCA1. Cancer Res. (2013) 73:1211–8. 10.1158/0008-5472.CAN-12-3128 23233737 PMC3563867

[17] Rysman E Brusselmans K Scheys K Timmermans L Derua R Munck S. De novo lipogenesis protects cancer cells fromfree radicals and chemotherapeutics by promoting membrane lipid saturation. Cancer Res. (2010) 70:8117–26. 10.1158/0008-5472.CAN-09-3871 20876798

[18] Baenke F Peck B Miess H Schulze A. Hooked on fat: the role of lipid synthesis in cancer metabolism and tumour development. Dis Model Mech. (2013) 6:1353–63. 10.1242/dmm.011338 24203995 PMC3820259

[19] Park J Lee C Jang J Ghim J Kim Y You S. Phospholipase signalling networks in cancer. Nat Rev Cancer. (2012) 12:782–92. 10.1038/nrc3379 23076158

[20] Chang S Liu C Conway R Han D Nithipatikom K Trifan O. Role of prostaglandin E2-dependent angiogenic switch in cyclooxygenase 2-induced breast cancerprogression. Proc Natl Acad Sci. (2004) 101:591–6. 10.1073/pnas.2535911100 14688410 PMC327192

[21] Ray G Husain S. Role of lipids, lipoproteins and vitamins in women with breast cancer. Clin Biochem. (2001) 34:71–6. 10.1016/S0009-9120(00)00200-9 11239519

[22] Shah F Shukla S Shah P Patel H Patel P. Significance of Alterations in Plasma Lipid Profile Levels in Breast Cancer. Integr Cancer Ther. (2008) 7:33–41. 10.1177/1534735407313883 18292593

[23] Ghahremanfard F Mirmohammadkhani M Shahnazari B Gholami G Mehdizadeh J. The Valuable Role of Measuring Serum Lipid Profile in Cancer Progression. Oman Med J. (2015) 30:353–7. 10.5001/omj.2015.71 26421116 PMC4576393

[24] Uzunlulu M Telci Caklili O Oguz A. Association between Metabolic Syndrome and Cancer. Ann Nutr Metab. (2016) 68:173–9. 10.1159/000443743 26895247

[25] Moore J Chaudhary N Akinyemiju T. Metabolic Syndrome Prevalence by Race/Ethnicity and Sex in the United States, National Health and Nutrition Examination Survey, 1988-2012. Prev Chronic Dis. (2017) 14:E24. 10.5888/pcd14.160287 28301314 PMC5364735

[26] Esposito K Chiodini P Colao A Lenzi A Giugliano D. Metabolic syndrome and risk of cancer: a systematic review and meta-analysis. Diabetes Care. (2012) 35:2402–11. 10.2337/dc12-0336 23093685 PMC3476894

[27] Saultier P Auquier P Bertrand Y Vercasson C Oudin C Contet A. Metabolic syndrome in long-term survivors of childhood acute leukemia treated without hematopoietic stem cell transplantation: an L.E.A. study. Haematologica. (2016) 101:1603–10. 10.3324/haematol.2016.148908 27515247 PMC5479621

[28] Ligibel J Alfano C Courneya K Denmark-Wahnefried W Burger R. American Society of Clinical Oncology Position Statement on Obesity and Cancer. J Clin Oncol. (2014) 32:3568–74. 10.1200/JCO.2014.58.4680 25273035 PMC4979237

[29] Avgerinos K Spyrou N Mantzoros C Dalamaga M. Obesity and cancer risk: Emerging biological mechanisms and perspectives. Metabolism. (2019) 92:121–35. 10.1016/j.metabol.2018.11.001 30445141

[30] Jaggers J Sui X Hooker S. Metabolic syndrome and risk of cancer mortality in men. Eur J Cancer. (2009) 45:1831–8. 10.1016/j.ejca.2009.01.031 19250819 PMC2700189

[31] Stebbing J Sharma A North B. A metabolic phenotyping approach to understanding relationships between metabolic syndrome and breast tumour responses to chemotherapy. Ann Oncol. (2012) 23:860–6. 10.1093/annonc/mdr347 21821546

[32] Lochhead P Chan A. Statins and colorectal cancer. Clin Gastroenterol Hepatol. (2013) 11:109–18. 10.1016/j.cgh.2012.08.037 22982096 PMC3703461

[33] Murai T. Cholesterol lowering: role in cancer prevention and treatment. Biol Chem. (2015) 396:1–11. 10.1515/hsz-2014-0194 25205720

[34] Pose E Trebicka J Mookerjee RP Angeli P Gines P. Statins: old drugs as new therapy for liver diseases? J Hepatol. (2019) 70:194–202. 10.1016/j.jhep.2018.07.019 30075229

[35] Zhang Y Liang M Sun C Qu G Shi T Min M. Statin Use and Risk of Pancreatic Cancer: An Updated Meta-analysis of 26 Studies. Pancreas. (2019) 48:142–50. 10.1097/MPA.0000000000001226 30640225

[36] Ahern T Lash T Christiansen P Cronin-Fenton D. Statins and breast cancer prognosis: evidence and opportunities. Lancet Oncol. (2014) 15:e461–8. 10.1016/S1470-2045(14)70119-6 25186049 PMC4167822

[37] Trotti A Colevas A Setser A. CTCAE. v3. 0: development of a comprehensive grading system for the adverse effects of cancer treatment. Semin Radiat Oncol. (2003) 13:176–81. 10.1016/S1053-4296(03)00031-6 12903007

[38] Tian W Yao Y Fan G. Changes in lipid profiles during and after (neo)adjuvant chemotherapy in women with early-stage breast cancer: a retrospective study. PLoS ONE. (2019) 14:e0221866. 10.1371/journal.pone.0221866 31465521 PMC6715243

[39] He T Wang C Tan Q. Adjuvant chemotherapy-associated lipid changes in breast cancer patients: a real-word retrospective analysis. Medicine (Baltimore). (2020) 99:e21498. 10.1097/MD.0000000000021498 32871996 PMC7437760

[40] Willemse PPM van der Meer R Burggraaf J van Elderen SGC de Kam ML de Roos A. Abdominal visceral and subcutaneous fat increase, insulin resistance and hyperlipidemia in testicular cancer patients treated with cisplatin-based chemotherapy. Acta Oncol. (2014) 53:351–60. 10.3109/0284186X.2013.819116 23957624

[41] Choi S Kam S. Metabolic effects of androgen deprivation therapy. Korean J Urol. (2015) 56:12. 10.4111/kju.2015.56.1.12 25598932 PMC4294850

[42] Crawford E Heidenreich A Lawrentschuk N Tombal B Pompeo AC Mendoza-Valdes A. Androgen-targeted therapy in men with prostate cancer: evolving practice and future considerations. Prostate Cancer Prostatic Dis. (2019) 22:24. 10.1038/s41391-018-0079-0 30131604 PMC6370592

[43] Torimoto K Samma S Kagebayashi Y Chihara Y Tanaka N Hirayama A. The Effects of Androgen Deprivation Therapy on Lipid Metabolism and Body Composition in Japanese Patients with Prostate Cancer. Jpn J Clin Oncol. (2011) 41:577–81. 10.1093/jjco/hyr005 21297122

[44] Salvador C Planas J Agreda F Placer J Trilla E Lopez M. Analysis of the Lipid Profile and Atherogenic Risk during Androgen Deprivation Therapy in Prostate Cancer Patients. Urol Int. (2013) 90:41–4. 10.1159/000342814 23235105

[45] Grossman M Zajac J. Management of side effects of androgen deprivation therapy. Endocrinol Metab Clin North Am. (2011) 40:655–71. 10.1016/j.ecl.2011.05.004 21889727

[46] Levine G D'Amico A Berger P Clark P Eckel R Keating N. Androgen-deprivation therapy in prostate cancer and cardiovascular risk: a science advisory from the American Heart Association, American Cancer Society, and American Urological Association: endorsed by the American Society for Radiation Oncology. Circulation. (2010) 121:833–40. 10.1161/CIRCULATIONAHA.109.192695 20124128 PMC3023973

[47] Estes NAM III Gersh BJ Hunt SA Otto CM UpToDate (2022). Available online at: https://www-uptodate-com.online.uchc.edu/contents/search (accessed January 6, 2022)

[48] Amir E Seruga B Niraula S Carlsson L Ocaña A. Toxicity of adjuvant endocrine therapy in postmenopausal breast cancer patients: a systematic review and meta-analysis. J Natl Cancer Inst. (2011) 103:1299–309. 10.1093/jnci/djr242 21743022

[49] Maor R Sara JDS Wanous AA Maor E Pruthi S Lerman A. Attenuated peripheral endothelial function among women treated with aromatase inhibitors for breast cancer. Coron Artery Dis. (2018) 29:687–93. 10.1097/MCA.0000000000000666 30379695

[50] Sharma M Tuaine J McLaren B. Chemotherapy Agents Alter Plasma Lipids in Breast Cancer Patients and Show Differential Effects on Lipid Metabolism Genes in Liver Cells. PLoS ONE. (2016) 11:148049. 10.1371/journal.pone.0148049 26807857 PMC4726544

[51] Zhou A Bai Y Song Y. Anlotinib Versus Sunitinib as First-Line Treatment for Metastatic Renal Cell Carcinoma: A Randomized Phase II Clinical Trial. Oncologist. (2019) 24:e702. 10.1634/theoncologist.2018-0839 30902918 PMC6693716

[52] Si X Zhang L Wang H. Management of anlotinib-related adverse events in patients with advanced non-small cell lung cancer: Experiences in ALTER-0303. Thorac Cancer. (2019) 10:551. 10.1111/1759-7714.12977 30666799 PMC6397894

[53] Han B Li K Wang Q. Effect of Anlotinib as a Third-Line or Further Treatment on Overall Survival of Patients With Advanced Non–Small Cell Lung Cancer: The ALTER 0303 Phase 3 Randomized Clinical Trial. JAMA Oncol. (2018) 4:1569. 10.1001/jamaoncol.2018.3039 30098152 PMC6248083

[54] Blais N Adam J Nguyen J Gregoire J. Evaluation and Management of Dyslipidemia in Patients Treated with Lorlatinib. Curr Oncol. (2021) 28:265–72. 10.3390/curroncol28010029 PMC7985771 36645013

[55] Bauer T Felip E Solomon B. Clinical Management of Adverse Events Associated with Lorlatinib. Oncologist. (2019) 24:1103. 10.1634/theoncologist.2018-0380 30890623 PMC6693708

[56] Verges B Walter T Cariou B. Endocrine Side Effects of Anti-Cancer Drugs: Effects of anti-cancer targeted therapies on lipid and glucose metabolism. Eur J Endocrinol. (2014) 170:R43–55. 10.1530/EJE-13-0586 24154684

[57] Hakeam HA Al-Jedai AH Raza SM Hamawi K. Sirolimus induced dyslipidemia in tacrolimus based vs. tacrolimus free immunosuppressive regimens in renal transplant recipients. Ann Transplant. (2008) 13:46–53. 18566560

[58] Morrisett J Abdel-Fattah G Hoogeveen R Morrisett JD Abdel-Fattah G Hoogeveen R. Effects of sirolimus on plasma lipids, lipoprotein levels, and fatty acid metabolism in renal transplant patients. J Lipid Res. (2002) 43:1170–80. 10.1194/jlr.M100392-JLR200 12177161

[59] Busaidy N Farooki A Dowlati A. Management of Metabolic Effects Associated With Anticancer Agents Targeting the PI3K-Akt-mTOR Pathway. J Clin Oncol. (2012) 30:2919. 10.1200/JCO.2011.39.7356 22778315 PMC3410405

[60] Dai H Liu C Li P. Risk of Dyslipidemia Associated with VEGF/VEGFR Inhibitors: a meta-analysis. Transl Oncol. (2020) 13:100779. 10.1016/j.tranon.2020.100779 32375082 PMC7205761

[61] Fishman M Srinivas S Hauke R. Phase Ib study of tivozanib (AV-951) in combination with temsirolimus in patients with renal cell carcinoma. Eur J Cancer. (2013) 49:2841. 10.1016/j.ejca.2013.04.019 23726267 PMC4666006

[62] Parsons S Skapek S Neufeld E. Asparaginase-Associated Lipid Abnormalities in Children With Acute Lymphoblastic Leukemia. Blood. (1997) 89:1886–95. 10.1182/blood.V89.6.1886 9058708

[63] Tozuka M Yamauchi K Hidaka H Nakabayashi T Okumura N Katsuyama T. Characterization of hypertriglyceridemia induced by L-asparaginase therapy for acute lymphoblastic leukemia and malignant lymphoma. Ann Clin Lab Sci. (1997) 27:351–7. 9303174

[64] Cohen H Bielorai B Harats D Toren A Pinhas-Hamiel O. Conservative treatment of L-asparaginase-associated lipid abnormalities in children with acute lymphoblastic leukemia. Pediatr Blood Cancer. (2010) 54:703–6. 10.1002/pbc.22305 20063421

[65] Bauters T Bordon V Laureys G Dhooge C. Combined use of ruxolitinib and sirolimus: increased monitoring of triglycerides required. Bone Marrow Transplant. (2019) 54:1372–3. 10.1038/s41409-019-0488-2 30804487

[66] Mesa R Verstovsek S Gupta V Mascarenhas JO Atallah E Burn T. Effects of Ruxolitinib Treatment on Metabolic and Nutritional Parameters in Patients With Myelofibrosis From COMFORT-I. Clin Lymphoma Myeloma Leuk. (2015) 15:214. 10.1016/j.clml.2014.12.008 25682576 PMC4418454

[67] Watson A Brunstein C Holtan S. Life-Threatening Hypertriglyceridemia in a Patient on Ruxolitinib and Sirolimus for Chronic Graft-versus-Host Disease. Case Rep Transplant. (2018) 2018:1–3. 10.1155/2018/4539757 30519495 PMC6241238

[68] Ligand, Pharmaceuticals Incorporated. Targretin® (bexarotene) [package insert]. U.S. Food and Drug Administration Website. Available online at: https://www.accessdata.fda.gov/drugsatfda_docs/label/1999/21055lbl.pdf

[69] de Vries-van der Weij J de Haan W Hu L Kuif M Oei HL van der Hoorn JWA. Bexarotene Induces Dyslipidemia by Increased Very Low-Density Lipoprotein Production and Cholesteryl Ester Transfer Protein-Mediated Reduction of High-Density Lipoprotein. Endocrinology. (2009) 150:2368–75. 10.1210/en.2008-1540 19147676

[70] Kurt M Babaoglu MO Yasar U Shorbagi A Guler N. Capecitabine-Induced Severe Hypertriglyceridemia: Report of Two Cases. Ann Pharmacother. (2006) 40:328–31. 10.1345/aph.1G348 16391007

[71] Duman B Paydas S Tetiker T Gunaldi M Afsar C Ercolak V. Capecitabine-Induced Hypertriglyceridemia and Hyperglycemia: Two Cases. Pharmacology. (2012) 90:212–5. 10.1159/000342382 23038659

[72] Arnett DK Blumenthal RS Albert MA Buroker AB Goldberger ZD Hahn EJ. 2019 ACC/AHA Guideline on the Primary Prevention of Cardiovascular Disease: A Report of the American College of Cardiology/American Heart Association Task Force on Clinical Practice Guidelines. J Am Coll Cardiol. (2019) 74:e177–232. 10.1016/j.jacc.2019.03.010 30894318 PMC7685565

[73] Lloyd-Jonse D Braun LT Ndumele CE Smith SC Jr Sperling LS Virani SS. Use of Risk Assessment Tools to Guide Decision-Making in the Primary Prevention of Atherosclerotic Cardiovascular Disease: a Special Report From the American Heart Association and American College of Cardiology. J Am Coll Cardiol. (2019) 73:3153–67. 10.1161/CIR.0000000000000638 30423392

[74] Pearson GJ Thanassoulis G Anderson TJ Stone J Ward R Wray W. 2021 Canadian Cardiovascular Society Guidelines for the Management of Dyslipidemia for the Prevention of Cardiovascular Disease in Adults. Soc Guidel. (2021) 37:1129–50. 10.1016/j.cjca.2021.03.016 33781847

[75] Chow EJ Chen Y Kremer L Breslow NE Hudson MM Armstrong GT. Individual prediction of heart failure among childhood cancer survivors. J Clin Oncol. (2015) 33:394–402. 10.1200/JCO.2014.56.1373 25287823 PMC4314592

[76] Strongman H Gadd S Matthews A Mansfield KE Stanway S Lyon AR. Medium and long-term risks of specific cardiovascular diseases in survivors of 20 adult cancers: a population-based cohort study using multiple linked UK electronic health records databases. Lancet. (2019) 394:1041–54. 10.1016/S0140-6736(19)31674-5 31443926 PMC6857444

[77] Armenian S Xu L Ky B Sun C Farol L Pal S. Cardiovascular Disease Among Survivors of Adult-Onset Cancer: A Community-Based Retrospective Cohort Study. J Clin Oncol. (2016) 34:1122–30. 10.1200/JCO.2015.64.0409 26834065 PMC7357493

[78] Garg P Jorgensen N McClelland R Leigh JA Greenland P Blaha M. Use of coronary artery calcium testing to improve coronary heart disease risk assessment in a lung cancer screening population: The Multi-Ethnic Study of Atherosclerosis (MESA). J Cardiovasc Comput Tomogr. (2018) 12:493–9. 10.1016/j.jcct.2018.10.001 30297128 PMC6585432

[79] Whelton S Al Rifai M Marshall CH Dardari Z Shaw LJ Al-Mallah M. Coronary Artery Calcium and the Age-Specific Competing Risk of Cardiovascular Versus Cancer Mortality: the Coronary Artery Calcium Consortium. Am J Med. (2020) 133:e575–83. 10.1016/j.amjmed.2020.02.034 32268145 PMC7541686

[80] Bahrami A Parsamanesh N Atkin SL Banach M Sahebkar A. Effect of statins on toll-like receptors: a new insight to pleiotropic effects. Pharmacol Res. (2018) 135:230–8. 10.1016/j.phrs.2018.08.014 30120976

[81] Green A Ramey DR Emneus M Iachina M Stavem K Bolin K. Incidence of cancer and mortality in patients from the Simvastatin and Ezetimibe in Aortic Stenosis (SEAS) trial. Am J Cardiol. (2014) 114:1518–22. 10.1016/j.amjcard.2014.08.016 25267716

[82] Savarese G De Ferrari G Rosano GM Perrone-Filardi P. Safety and efficacy of ezetimibe: a meta-analysis. Int J Cardiol. (2015) 201:247–52. 10.1016/j.ijcard.2015.08.103 26301648

[83] Zhan S Tang M Liu F Xia P Shu M Wu X. Ezetimibe for the prevention of cardiovascular disease and all-cause mortality events. Cochrane Database Syst Rev. (2018) 11:CD012502. 10.1002/14651858.CD012502.pub2 30480766 PMC6516816

[84] Ferreira AM da Silva PM. Defining the Place of Ezetimibe/Atorvastatin in the Management of Hyperlipidemia. Am J Cardiovasc Drugs. (2017) 17:169–81. 10.1007/s40256-016-0205-0 27943172

[85] Page M Watts G. PCSK9 inhibitors – mechanisms of action. Aust Prescr. (2016) 39:164. 10.18773/austprescr.2016.060 27789927 PMC5079795

[86] Liu X Bao X Hu M Chang H Jiao M Cheng J. Inhibition of PCSK9 potentiates immune checkpoint therapy for cancer. Nature. (2020) 588:693–8. 10.1038/s41586-020-2911-7 33177715 PMC7770056

[87] Wei S Espenshade P. Lipids: Cholesterol Synthesis and Regulation. In: Encyclopedia of Biological Chemistry III. Wei S, Espenshade P. Lipids: Cholesterol Synthesis and Regulation. In: *Encyclopedia of Biological Chemistry III. Third*. (2021) p. 732–8. Available at: https://www.sciencedirect.com/science/article/pii/B9780128194607000219

[88] Di Minno A Lupoli R Calcaterra I Poggio P Forte F Spadarella G. Efficacy and Safety of Bempedoic Acid in Patients With Hypercholesterolemia: Systematic Review and Meta-Analysis of Randomized Controlled Trials. J Am Heart Assoc. (2020) 9:e016262. 10.1161/JAHA.119.016262 32689862 PMC7792250

[89] Bhatt DL Steg PG Miller M Brinton EA Jacobson TA Ketchum SB. Cardiovascular Risk Reduction with Icosapent Ethyl for Hypertriglyceridemia. N Engl J Med. (2019) 380:11–22. 10.1056/NEJMoa1812792 30415628

[90] McAlister R Aston J Pollack M Du L Koyama T Chism D. Effect of Concomitant pH-Elevating Medications with Pazopanib on Progression-Free Survival and Overall Survival in Patients with Metastatic Renal Cell Carcinoma. Oncologist. (2018) 23:686–92. 10.1634/theoncologist.2017-0578 29487220 PMC6067930

[91] Haouala A Widmer N Duchosal MA Montemurro M Buclin T Decosterd LA. Drug interactions with the tyrosine kinase inhibitors imatinib, dasatinib, and nilotinib. Blood. (2011) 117:e75–387. 10.1182/blood-2010-07-294330 20810928

[92] Thompson PD Panza G Zaleski A Taylor B. Statin-Associated Side Effects. J Am Coll Cardiol. (2016) 67:2395–410. 10.1016/j.jacc.2016.02.071 27199064

[93] Schierwagen R Uschner FE Magdaleno F Klein S Trebicka J. Rationale for the use of statins in liver disease. Am J Physiol Gastrointest Liver Physiol. (2017) 312:G407–12. 10.1152/ajpgi.00441.2016 28280144

[94] Cavallo J,. Building Onco-Primary Care to Close the ‘Black Hole' in Cancer Survivorship Care A Conversation With Kevin Oeffinger, MD. The ASCO Post (2020). Available online at: https://ascopost.com/issues/march-10-2020/building-onco-primary-care-to-close-the-black-hole-in-cancer-survivorship-care/ (cited March 26, 2022).

